# Novel *sul*I binary vectors enable an inexpensive foliar selection method in *Arabidopsis*

**DOI:** 10.1186/1756-0500-4-44

**Published:** 2011-03-02

**Authors:** James G Thomson, Meridith Cook, Mara Guttman, Jamison Smith, Roger Thilmony

**Affiliations:** 1 USDA-ARS, Western Regional Research Center, Crop Improvement and Utilization Research Unit, 800 Buchanan Street, Albany, CA 94710 USA

## Abstract

**Background:**

Sulfonamide resistance is conferred by the *sul*I gene found on many *Enterobacteriaceae *R plasmids and Tn21 type transposons. The *sul*I gene encodes a sulfonamide insensitive dihydropteroate synthase enzyme required for folate biosynthesis. Transformation of tobacco, potato or *Arabidopsis *using *sul*I as a selectable marker generates sulfadiazine-resistant plants. Typically *sul*I-based selection of transgenic plants is performed on tissue culture media under sterile conditions.

**Findings:**

A set of novel binary vectors containing a *sul*I selectable marker expression cassette were constructed and used to generate transgenic *Arabidopsis*. We demonstrate that the *sul*I selectable marker can be utilized for direct selection of plants grown in soil with a simple foliar spray application procedure. A highly effective and inexpensive high throughput screening strategy to identify transgenic *Arabidopsis *without use of tissue culture was developed.

**Conclusion:**

Novel *sul*I-containing *Agrobacterium *binary vectors designed to over-express a gene of interest or to characterize a test promoter in transgenic plants have been constructed. These new vector tools combined with the various beneficial attributes of sulfonamide selection and the simple foliar screening strategy provide an advantageous alternative for plant biotechnology researchers. The set of binary vectors is freely available upon request.

## Background

Genetic transformation is a valuable tool in plant research and crop improvement. Selectable marker systems are integral components facilitating the selection and identification of transformed cells [1]. Although *in planta *transformation of *Arabidopsis *is a relatively simple and efficient process [2,3] a versatile, non-labor intensive strategy enabling the straightforward identification of the transformed plants is still desirable. Commonly used selectable marker genes for plant transformation include the *neomycin phosphotransferase *II gene (*npt*II) [4-6] that confers resistance to aminoglycoside antibiotics such as kanamycin, neomycin, and G418, as well as the *hygromycin phosphotransferase *II gene (*hpt*II) [7] and *bialaphos resistance *gene (*bar*) [8] that confer resistance to hygromycin and the herbicide glufosinate, respectively. A lesser known and less frequently utilized plant selectable marker is the *sulfonamide resistance *gene (*sul*I), which confers resistance to sulfadiazine and other sulfonamide chemicals [9]. Since the first demonstration of its utility in tobacco, others have utilized *sul*I for selection in potato [10,11] as well as *Arabidopsis *[12,13]. Arguably, *sul*I is used less often than *npt*II, *hpt*II, and *bar *simply due to its infrequent inclusion as a marker in the commonly used plant transformation vectors. However, several attributes of a *sul*I selection strategy support its potential for increased use, including the stability and low human toxicity of sulfonamide chemicals, as well as their cost effectiveness. Sulfadiazine is significantly less expensive than either kanamycin or hygromycin. Sulfonamides also exhibit substantial potency as a plant selection agent; e.g. 5 mg/L sulfadiazine is sufficient for *Arabidopsis *selection [12].

Sulfadiazine and other sulfonamides are inhibitors of the enzyme dihydropteroate synthase (DHPS) (EC 2.5.1.15). This enzyme is part of the folic acid metabolic pathway present in microbes and plants (but not human cells) and catalyzes the formation of the intermediate dihydropteroic acid [14,15]. Folic acid is required for a number of cellular processes including the biosynthesis of purine nucleotides and metabolism of the amino acids serine, glycine, histidine and methionine. The selective action of sulfonamide chemicals is the competitive inhibition of DHPS, which blocks folate biosynthesis in the cells [16]. Sulfadiazine is a structural analog to *p*-aminobenzoic acid, the normal substrate, and can function as an alternative substrate producing a non-functional sulfa-containing pteroate analog inhibiting the pathway [15]. The *sul*I resistance gene from the R46 plasmid of *E. coli *encodes a DHPS that is insensitive to sulfonamides, and thus continues folate synthesis in the presence of sulfadiazine.

Selection of transgenic *Arabidopsis *with the antibiotics kanamycin or hygromycin is generally limited to screening of surface sterilized seed germinated under aseptic conditions in tissue culture and thus requires substantial effort, as well as specialized equipment and materials. Selection of transgenic *Arabidopsis *containing the *bar *gene can be performed either in culture with medium containing bialaphos or phosphinothricin, or can be performed on plants grown in soil using foliar application of glyphosinate herbicides [17]. To facilitate plant biotechnology research, we were interested in identifying an alternative selection strategy that was inexpensive and versatile enough for high-throughput screening of soil grown plants as well as for screening plants grown in tissue culture.

Here we present the construction and validation of several novel *Agrobacterium *binary vectors containing a *sul*I selectable marker cassette. These vectors can facilitate either the over-expression of candidate genes or the characterization of candidate promoters in transgenic *Arabidopsis *and potentially other dicot plants. In addition, we have developed a simple, inexpensive strategy for screening T_1 _plants grown in soil using a foliar spray of sulfadiazine that allows efficient, high-density selection and identification of transgenic *Arabidopsis*.

## Materials and methods

### Plasmid Constructs

**Figure 1:**
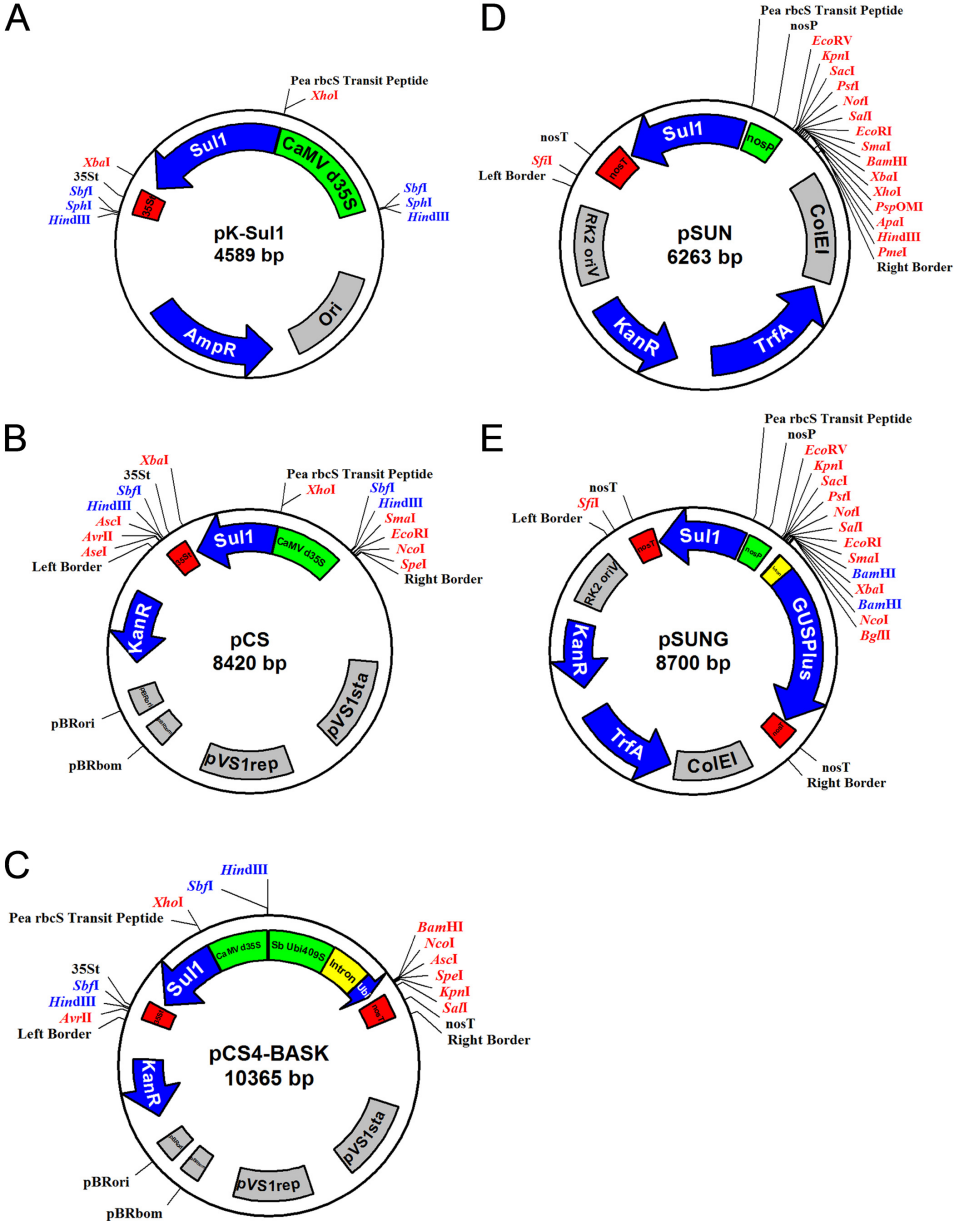
**Schematic diagrams of five *sul*I vectors**. (A) Plasmid map of the pK-*Sul*I construct; a pUC-derived vector that contains the CaMV double enhanced 35S promoter (CaMV d35S) fused to the *sul*I coding sequence with the CaMV 35S terminator (35St). (B) Map of the pCS binary vector containing the CaMV d35S-*sul*I-35St cassette derived from pK-SulI. (C) Map of the pCS4-BASK constitutive expression construct. In addition to the db35S driven *sul*I selection gene, the T-DNA contains the *Solanum bulbocastanum *409s promoter (Sb Ubi409s), intron and first ubiquitin monomer coding sequence and a nos 3' terminator (nosT). A translational fusion to the ubiquitin monomer (Ubi) is required for overexpression. (D) Map of the pSUN binary vector which carries a nopaline synthase gene promoter (nosP) and terminator (nosT) to express the *sul*I selectable marker within the T-DNA. (E) Map of the pSUNG promoter testing construct, a pSUN-derived vector with a promoterless *GUSPlus *coding sequence and nos terminator located near the T-DNA right border. Unique and/or potentially useful restriction sites are shown. Gene coding sequences are drawn as blue arrows, promoters as green boxes, 3' terminators/poly adenylation signals as red boxes, introns as yellow boxes and the vector origin or replication sequences as gray boxes. Vector maps are not drawn to scale. Plasmid sequences are available from GenBank, accession numbers: HQ593859 - HQ593863.

The pK-*sul*I, pCS4-BASK, pSUN and pSUNG plasmid vectors (Figure 1) and their derivatives utilized in this study are described below. All plasmids were introduced into bacteria using either a RbCl_2 _heat shock transformation method or via electroporation [18].

### *pK-Sul*I

The *sul*I coding sequence including a truncated pea chloroplast transit peptide was PCR amplified (Phusion, NEB) from pJIT119 [9] using primers (Pea STD *Xho*I F61 5'-agtcCTCGAGatgggcccattcggcggc-3' and *sul*I *Xba*I R60 5'-agtcTCTAGActaggcatgatctaaccctcggtctc-3'). The amplicon was cloned between the CaMV double 35S promoter-35S terminator elements of the pKar6 vector (Robert Blanvillain and Patrick Gallois, unpublished data) using the *Xho*I and *Xba*I sites. This provided a highly expressed *sul*I gene cassette that is flanked by *Hind*III, *Sph*I and *Sbf*I sites for ease of subcloning (Figure 1A).

### pCS construction

The pCAMBIA 390 binary vector (http://www.cambia.org) was modified by removing the pre-existing Nos3 terminator and *Agrobacterium *Right Border with a *Spe*I and *Sph*I digestion. The *Agrobacterium *Right Border region was PCR amplified (Phusion, NEB) using primers (RB SpeI F61 5'-aactACTAGTgtttgacaggatatattggc-3' and RB SphI R62 5'-aacGCATGCgaagccgactgcac-3'). The amplicon was *Spe*I - *Sph*I digested and reintroduced into pCAMBIA 390A, thereby removing the Nos3 terminator and reforming the *Agrobacterium *Right Border. Finally, the pK-*Sul*I cassette (described above) was cloned into the *Hind*III site oriented towards the Left Border as shown in Figure 1B.

### pCS4-BASK construction

The *Solanum bulbocastanum *409s promoter was PCR amplified (Phusion, NEB) from pBINPLUS/ARS-409S [19] and cloned into the pACH20 vector [20] between the *Hind*III and *Bam*HI sites. The *bar *coding sequence was excised by a *Bam*HI and *Kpn*I digestion and a *Bam*HI/*Asc*I/*Spe*I/*Kpn*I (BASK) multiple cloning site inserted in its place. The pCAMBIA 390 binary vector was modified by removing the pre-existing *Asc*I site by digestion and fill in with Klenow to create pCAMBIA 390A. The 409s-BASK-Nos3 terminator cassette was excised from the pUC backbone by *Hind*III and *Eco*RI digestion and cloned into pCAMBIA 390A (described above). This resulted in a LB-409s-BASK-Nos3-Nos3-RB configuration due to the presence of a pre-existing Nos3 terminator within the pCAMBIA 390A vector. The extra Nos3 terminator was removed by *Mau*BI digest and religation. Finally, the pK-*Sul*I cassette (described above) was cloned into the *Hind*III site in an inverted orientation relative to the 409s-BASK-Nos3 terminator expression cassette (Figure 1C).

### pCS4-GFP construction

The *GFP *coding sequence was PCR amplified (Phusion High-Fidelity DNA Polymerase, New England Biolabs) using primers (GFP BHI F60 5'- agtcGGATCCatggtgagcaagggcgagg-3' and GFP *Spe*I R60 5'-agtcACTAGTtcagcgagctctagggcc-3') using the pIRES-EGFP (Clontech) construct as the template. The amplicon was digested and ligated into the *Bam*HI and *Spe*I sites of pCS4-BASK. Sequence analysis confirmed that *GFP *was translationally fused to the ubiquitin monomer of the 409s expression cassette.

### pSUN construction

The selectable marker cassette within the T-DNA region of pORE-O3 [21] was removed by digestion with *Eco*RV and *Avr*II. The *Avr*II site was blunted using Klenow and an *Eco*RV flanked nos promoter-*sul*I-nos terminator cassette from a pGreen series vector [22] was inserted to create pSUN. The nos-*sul*I cassette is oriented towards the Left Border as shown in Figure 1D.

### pSUNG construction

A promoterless reporter gene cassette was added to the pSUN vector. A *GUSPlus *coding sequence with a nos terminator from pCAMBIA1305.1 (http://www.cambia.org) was excised and cloned into the pUC19 vector, then subcloned into pSUN with *Hind*III and *Xba*I. The promoterless *GUSPlus *cassette is located near the Right Border within the T-DNA and is separated from the *sul*I selection marker cassette by a multiple cloning site as shown in Figure 1E.

### Sulfadiazine Selection of Transgenic Plants in Culture

Seeds were collected in bulk from plants infiltrated with *Agrobacterium *harboring one of the previously described constructs. Dry seeds were either sown directly in soil or surface sterilized and placed on 150 mm agar plates containing 1 × MS salts plus B5 vitamins solidified with 2.0 g/L Bacto Agar as described [2], supplemented with 1 to 200 mg/L sulfadiazine (S8626, Sigma-Aldrich, St. Louis, MO).

### Arabidopsis thaliana growth and transformation

*Arabidopsis thaliana *Col-0 and L*er *ecotypes were used throughout this study. Seeds were stratified at 4°C for 2 days to synchronize germination and then grown in Sunshine mix #1 (SunGrow Horticulture Distribution, Bellevue WA) in a greenhouse or on media in a growth chamber with a 16 hour photoperiod, at constant temperature of 22°C and a light intensity of ~50 mE/m^2^/s. Plants were transformed using the floral dip method [2] with *Agrobacterium tumefaciens *strain GV3101.

### Sulfadiazine Foliar Application

For spray selection, seeds were sown in soil and germinated under a dome to maintain high humidity. Seedlings at the cotyledon stage were uncovered and sprayed to fully wet the plants and surrounding soil with a solution containing 0.03% L-77 silwet and sulfadiazine at concentrations ranging from 50 to 500 mg/L. Following spraying, the seedlings were re-covered for 24-48 hours with a plastic dome to maintain high humidity. The sulfadiazine spray was reapplied similarly every third day for a total of one to four applications.

### Cc1 promoter isolation and pSUNG-OsCc1 construction

A 1.8-kb fragment of the rice *Cytochrome c *gene (*OsCc1*) promoter was PCR-amplified (Phusion High-Fidelity DNA Polymerase, New England Biolabs) from *Oryza sativa japonica *cv. Nipponbare genomic DNA using a forward primer including a *Bam*HI site (*OsCc1*promFor1: 5'-aaGGATCCgagatcttcgaaggtaggc-3') and reverse primer including an *Nco*I site (*OsCc1*promRev1: 5'-aaCCATGGccgccgccgccgcgagaacg-3'). The *OsCc1 *promoter sequence from *Oryza sativa indica *drives constitutive expression in transgenic rice [23]. The *OsCc1 *promoter used in our analyses is 80% identical to the *indica *sequence. pSUNG was digested with *Bam*HI and *Nco*I, and the *OsCc1 *promoter was inserted upstream of *GUSPlus *forming a transcriptional fusion and generating the pSUNG-OsCc1 vector. pSUNG-OsCc1 was transformed into *Arabidopsis thaliana *ecotype Col-0 via floral dipping with *Agrobacterium tumefaciens*. Seeds were selected on media containing 25 mg/L sulfadiazine. Resistant seedlings were cultivated in soil. T_2 _generation seeds were selected on media containing 25 mg/L sulfadiazine and histochemically stained for β-glucuronidase activity using a standard protocol [24].

### PCR analysis

Genomic DNA was extracted by grinding a single leaf in 400 μl of buffer (200 mM Tris HCl pH 7.5, 250 mM NaCl, 25 mM EDTA, 0.5% SDS). After centrifugation, the isopropanol precipitated pellet was washed with 70% ethanol and resuspended in 50 μl of water. Two μl of genomic DNA in a 25 μl volume was used per PCR reaction. Primers used were: *sul*I F60 5'-atggtgacggtgttcggcattc-3' and *sul*I R60 5'-ctaggcatgatctaaccctcggtctc-3'; *hpt*II F62 5'-ggtgtcacgttgcaagacct-3' and *hpt*II R62 5'-cgtctgctgctccatacaag-3'; *npt*II F57 5'-gattgaacaagatggattgcacgc-3' and *npt*II R58 5'-ccacagtcgatgaatccagaaaagc-3'.

### Southern blot analysis

Genomic DNA was isolated using a rapid protocol [25] from randomly picked plants that survived sulfadiazine selection. The DNA samples were digested with *Hind*III, blotted to nylon membrane, and probed with a ^32^P-labeled *sul*I sequence amplified with the *sul*I F60 and *sul*I R60 primers described above. Probe hybridization was accomplished following the Rapid Hyb (Amersham) protocol. The membranes were washed at high stringency in 0.5XSSC and 0.1% SDS at 65°C for 15 minutes before exposing to X-ray film.

## Results and Discussion

### Construction and characterization of the *sul*I plasmid vectors

Several novel vectors have been constructed that utilize a *sul*I selectable marker cassette. The pK-*Sul*I cloning vector [GenBank:HQ593859] contains a double enhanced CaMV 35S promoter and terminator *sul*I expression fragment flanked by convenient restriction sites (*Hind*III, *Sph*I and *Sbf*I) that facilitate subcloning (Figure 1A). Four *Agrobacterium *binary vectors useful for plant transformation have also been generated. The pCS4-BASK vector [GenBank:HQ593861] contains the 35S *sul*I cassette from pK-*Sul*I and a *Solanum bulbocastanum *Ubi 409s promoter, intron and ubiquitin monomer [19] separated by a multiple cloning site from a nos terminator sequence (Figure 1C). This construct is useful for the strong over-expression of a gene of interest as a translational fusion to the 409s ubiquitin monomer.

The pSUNG binary vector [GenBank:HQ593863] was also constructed. This plasmid is well suited for investigating the function of candidate promoter sequences. The pSUNG construct contains a promoterless *GUSPlus *reporter gene with a large multiple cloning site upstream and a nos promoter-*sul*I-nos terminator selectable marker (Figure 1E). The nos promoter was utilized in this vector to minimize undesirable interactions between the selectable marker promoter and the candidate promoter being tested. The CaMV 35S promoter/enhancer is known to promiscuously interact with nearby promoters in transgenic *Arabidopsis*, confounding reporter gene expression characterization studies [26-28]. Further analysis has demonstrated that the nos promoter, in contrast, does not alter the expression conferred by nearby promoters [27,28] and thus was chosen as the promoter to control *sul*I expression in pSUNG.

In addition to the construction of the pCS4-BASK and pSUNG vectors, we also developed the precursor vectors pCS [GenBank:HQ593860] and pSUN [GenBank:HQ593862], respectively. These binary plasmids carry only a *sul*I selectable marker cassette and a multiple cloning site within the T-DNA. The maps of these adaptable 'empty' vectors are shown in Figures 1B and 1D. Although these vectors are already useful tools for generating plant transformation constructs, additional utility may be added in the future by generating Gateway^®^-compatible (Invitrogen) versions, which will enable high throughput cloning applications.

### Validation of the *sul*I selection constructs

To demonstrate the utility of these novel vectors, numerous transgenic *Arabidopsis *plants were generated via *Agrobacterium*-mediated floral dip transformation. Initially the functionality of the *sul*I selectable marker was examined. Sulfadiazine screening was performed in tissue culture and multiple independent transgenic plant lines were identified for each construct. The results demonstrate that both the 35S expression cassette of the pCS-derived vectors and the nos promoter expression cassette present on the pSUN-derived constructs successfully generate transgenic plants resistant to 5 to 50 mg/L sulfadiazine when germinated in culture. To determine how effectively the pCS- and pSUN-derived vectors confer resistance, the initially isolated lines were subjected to a concentration gradient of 0 - 200 mg/L sulfadiazine. As expected from previous results [12], the pCS-derived plants exhibited resistance and grew well in media containing up to 200 mg/L sulfadiazine. The pSUN-derived plants however were less tolerant, exhibiting good growth at levels up to 50 mg/L, with a gradual loss of fitness at higher levels of selection (data not shown). Even though the pSUN plants exhibit a more modest level of resistance than the pCS-derived plants, the observed resistance in the nos-*sul*I transgenics is sufficient to provide a clearly distinct resistance phenotype compared to wildtype plants at levels from 5 to 50 mg/L sulfadiazine.

**Figure 2:**
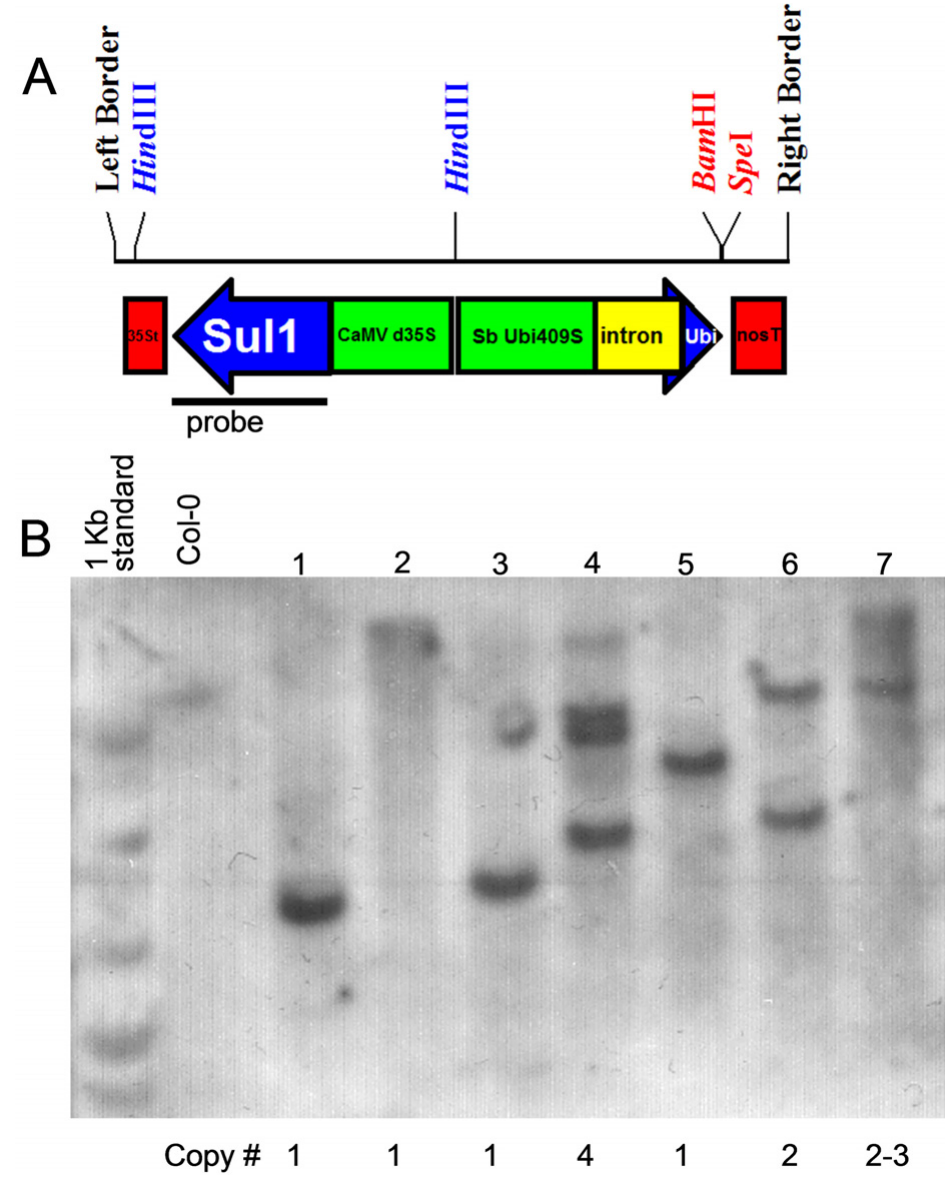
**Southern blot analysis of pCS4-BASK (*sul*I) transgenic plants**. (A) Schematic representation of CS4-BASK T-DNA where *Hind*III, *Bam*HI and *Spe*I endonuclease sites are shown. The *sul*I hybridization probe is indicated by thick line. (B) Genomic DNA from seven randomly chosen sulfadiazine-resistant plants and a wildtype Col-0 plant were digested with *Bam*HI and hybridized with a ^32^P labeled *sul*I probe. The estimated T-DNA copy number in each plant is shown below the lane.

Southern blot analysis examining the T-DNA copy number of seven independent pCS4-BASK transgenic plants using the *sul*I sequence as a probe was performed and the result is shown in Figure 2. Four of the seven plants exhibit a single uniquely sized band suggesting that they each contain a distinct T-DNA insertion, while three other plants exhibited two to four bands and likely carry two or more T-DNAs. Taken together, these results support the conclusion that a single copy of the *sul*I selection marker in the pCS- and pSUN-derived vectors is sufficient for selecting transgenic plants.

### Sulfadiazine foliar application

**Figure 3:**
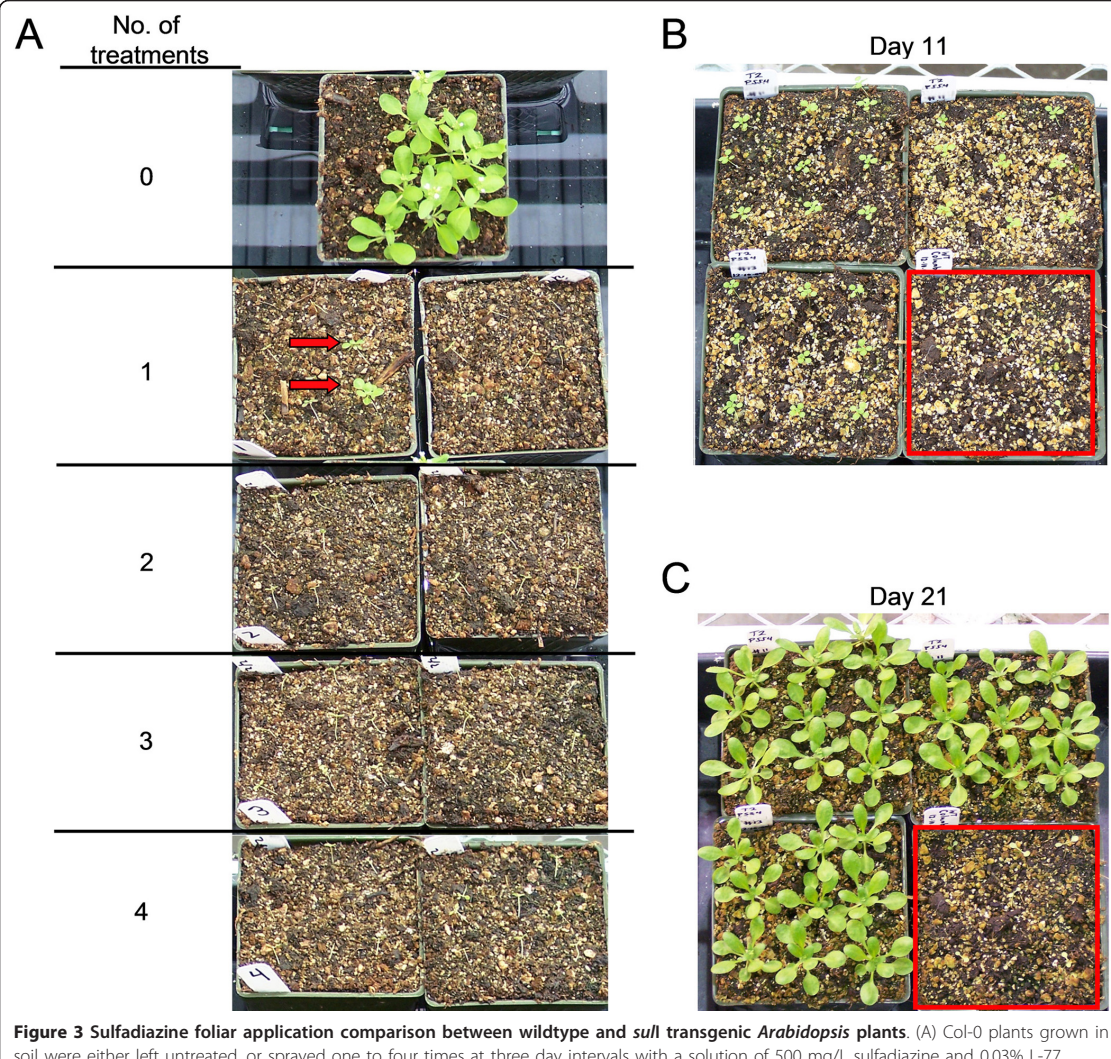
**Sulfadiazine foliar application comparison between wildtype and *sul*I transgenic *Arabidopsis *plants**. (A) Col-0 plants grown in soil were either left untreated, or sprayed one to four times at three day intervals with a solution of 500 mg/L sulfadiazine and 0.03% L-77 silwet as indicated. Plant growth was photographed 21 days following the first sulfadiazine application. Partially stunted plants are marked with red arrows in the sample sprayed once. (B) The growth of three independent pCS4-BASK transgenic plant lines and the wildtype control plants (red box) 11 days after the first spraying with a 500 mg/L sulfadizine solution. (C) Plant growth observed at 21 days after the initial sulfadiazine application.

Although our results and those of others have established that a tissue culture germination screen is an effective approach for identifying *sul*I transgenic *Arabidopsis*, a potential alternative screening method was also investigated. The use of foliar application of a sulfadiazine solution supplemented with 0.03% of the surfactant L-77 silwet was assessed. Col-0 plants were grown directly in soil and sprayed with 50, 100 and 500 mg/L sulfadiazine solution every three days for a total of four applications. Plants from the 50 and 100 mg/L treatment were partially stunted but continued to grow normally once the treatment ceased (data not shown). However, the Col-0 wildtype plants that experienced four applications at 500 mg/L were severely stunted (i.e. true leaves never emerged) and did not recover once the treatment was discontinued (data not shown). To further examine whether the selective pressure of multiple applications of 500 mg/L sulfadiazine was necessary to inhibit growth, wildtype plants were sprayed with the sulfadiazine solution either one, two, three or four times at three day intervals. As shown in Figure 3A, the wildtype Col-0 plants exhibited stunted growth and subsequent chlorosis even after a single treatment, although a few plants continued to grow and develop. However, the results from two to four applications demonstrate that multiple foliar treatments with sulfadiazine are extremely effective at inhibiting the growth of *Arabidopsis *seedlings in soil.

The resistance to the foliar application of sulfadiazine was examined in several confirmed *sul*I transgenic plants. Seed from three independent pCS4-BASK and pSUN homozygous transgenic lines were sown in soil and treated with a 500 mg/L sulfadiazine foliar spray. Figure 3B and 3C illustrates that the pCS4-BASK transgenic plants were fully resistant to sulfadiazine and exhibited normal growth at 11 and 21 days after the initial treatment. The initial sign of sulfadiazine resistance under these conditions is the emergence of the first set of true leaves, as is visible at day 11 (Figure 3B). Similar to the more modest level of resistance observed in tissue culture for the pSUN-derived plants, we observed that some of the pSUN plants exhibited less resistance to the foliar application of sulfadiazine as well. We speculate that this is likely due to the nos promoter driving lower levels of *sul*I expression in the transgenic plants than is typically observed for the double enhanced CaMV 35S promoter. Although we believe this is a likely explanation, other potential causes for this difference are also possible.

These results demonstrate that repeated foliar application of the sulfadiazine/silwet solution does not cause tissue damage to *sul*I resistant plants and can be successfully used to identify transgenic *Arabidopsis*. Under this foliar selection procedure, wildtype plants exhibit stunted growth but remain viable up to 14 days after the initial application, but by the end of three weeks, these plants are chlorotic and severely under-developed. In contrast, *sul*I transgenic plants continue to grow throughout subsequent treatments and are robust and healthy after three weeks. In fact, the *sul*I transgenic plants were frequently larger and healthier than even unselected wildtype plants grown side-by-side under the same conditions in the greenhouse.

**Figure 4:**
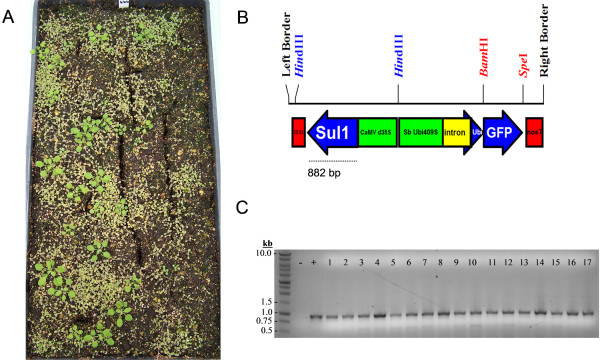
**Sulfadiazine foliar spray selection of T_1 _pCS4-GFP transgenic plants grown at a high density in soil**. Sulfadiazine solution (500 mg/L with 0.03% L-77 silwet) was applied every third day for a total of three applications. A) Plant growth 21 days after the initial sulfadiazine application. B) Schematic representation of the CS4-GFP T-DNA, where the dashed line represents the region PCR amplified with *sul*I primers. C) PCR amplification results confirming the presence of *sul*I in 17 randomly chosen sulfadiazine resistant plants. The dash (-) indicates a water negative control, and the plus (+) indicates a plasmid positive control. A size standard labeled in kilobase pairs (kb) is shown.

A method of three foliar applications was chosen to screen a dense population of *Arabidopsis *seedlings (approximately 4,000 per flat) germinated in soil containing a mixture of nontransgenic plants with candidate pCS4-GFP T_1 _individuals generated from *Agrobacterium*-mediated floral dip transformation. Following germination, the seedlings were sprayed three times at three day intervals with a 500 mg/L sulfadiazine/0.03% L-77 silwet solution. Plant growth at day 21 is shown in Figure 4A. Clearly, there are numerous plants that exhibit tolerance to sulfadiazine, while the majority are substantially stunted and chlorotic. The frequency of resistant plants (approximately 1.0%) is well within the range expected for a typical transformation and selection. Genomic DNA was isolated from the plants that were judged sulfadiazine resistant and subsequently screened with *sul*I-specific primers. Thirty-eight of the 39 individuals screened were confirmed to contain the *sul*I sequence verifying that the foliar screening of soil grown plants is effective and allows few if any non-transgenic plants to escape selection (Figure 4C). The nontransgenic plant that survived selection appeared only weakly resistant to sulfadiazine and was one of the smallest surviving plants. If desired, the stringency of the selection could be heightened by increasing the number of foliar applications and/or by choosing only the largest healthiest plants, although this strategy would likely overlook transgenic plants with marginal levels of sulfadiazine resistance. To examine how the *sul*I-based selection compared to the *npt*II and *hpt*II selectable markers, we germinated T_1 _seed on media containing 50 μg/ml kanamycin and 20 μg/ml hygromycin, respectively. Based on several independent experiments, we observed selection escape rates that ranged from 0% to 36% for kanamycin and 2% to 23% for hygromycin (data not shown). Taken together, these results illustrate that *sul*I foliar selection in soil performs at least as well as selection with other frequently used screening methods.

### Validation of the overexpression and promoter testing capabilities of the binary vectors

**Figure 5:**
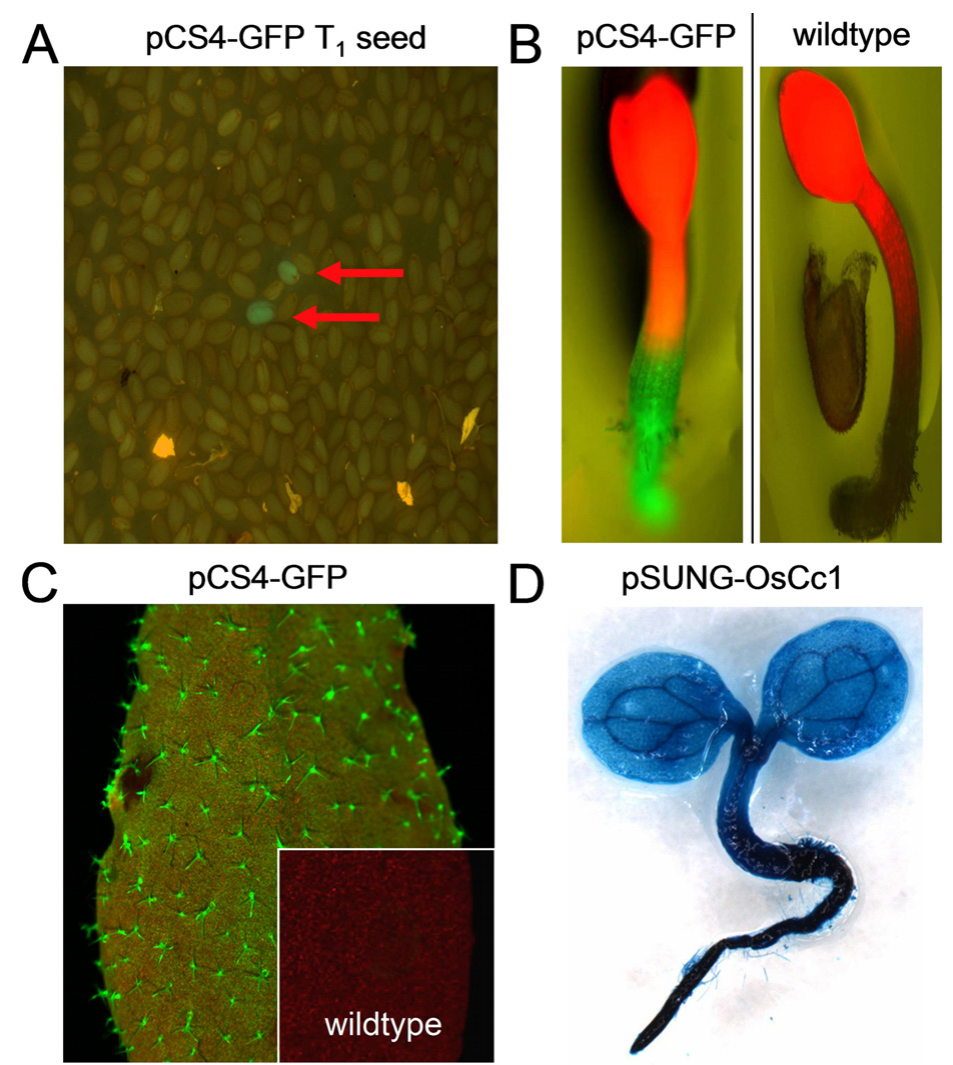
**Reporter gene activity observed in *sul*I transgenic plants**. (A) Green fluorescence observed in pCS4-GFP T_1 _seeds excited with blue light. Fluorescent seeds are marked with arrows. (B) Four-day-old pCS4-GFP and Col-0 seedlings visualized under blue light. (C) An adult leaf from a pCS4-GFP transgenic plant observed under blue light. The inset image shows the red autofluorescence observed in a nontransgenic leaf. (D) Histochemical GUS staining of a seven-day-old pSUNG-OsCc1 transgenic *Arabidopsis *seedling selected on medium containing 25 mg/L sulfadiazine.

To characterize the pCS4-BASK and pSUNG vectors, we examined the functionality of the other components within the T-DNA. An enhanced *GFP *coding sequence was translationally fused downstream of the ubiquitin monomer in pCS4-BASK and transformed into *Arabidopsis*. As shown in Figure 5A, B and 5C, the 409s promoter conferred strong GFP expression that is clearly visible in dry seeds, in young seedlings and in the trichomes of mature leaves. This demonstrates that the pCS4-BASK construct offers a viable alternative to using the 35S promoter for the over-expression of genes in *Arabidopsis *and likely other dicot plant species.

To validate the utility of the reporter gene in pSUNG, we fused a *japonica *rice *Cytochrome c *(*OsCc1*) promoter to *GUSPlus *to generate the pSUNG-OsCc1 vector. The *indica OsCc1 *promoter confers constitutive reporter gene expression in transgenic rice [23]. Figure 5D illustrates the GUSPlus reporter expression controlled by the *OsCc1 *promoter in a transgenic *Arabidopsis *seedling. *OsCc1 *transgenic plants exhibited strong GUS histochemical staining in all the tissues of young seedlings including both the root and the shoot. Weaker staining was observed in mature leaves (data not shown). These results demonstrate that the reporter gene portion of the pSUNG vector functions as expected and validates the utility of this novel construct for promoter testing. In addition, it provides evidence that the rice *OsCc1 *promoter not only controls strong expression in rice, but *Arabidopsis *seedlings as well and thus may be a useful promoter for expression in a diverse array of species.

The rapid development of genetics and the desire to answer more complicated questions often demand the simultaneous use of two or more selectable markers. We investigated the feasibility of stacking *sul*I with other selectable markers. As such, we chose to cross *sul*I transgenic plants with a previously generated *Arabidopsis *line that was homozygous for both the *npt*II and *hpt*II selectable markers [29]. Additional file 1 shows germinated seeds from the crossed plants screened on media supplemented with sulfadiazine (5 mg/L), hygromycin (10 mg/L), and kanamycin (50 mg/L). The triple transgenic seedlings grew normally, but the Col-0 wildtype failed to survive past the emergence of the radicle. These results indicate that simultaneous expression of the *sul*I, *npt*II and *hpt*II genes is not detrimental to the health of the transgenic plants, and that a triple selection screen is feasible, since the three antibiotics can be successfully used together in selection media.

## Conclusions

A novel foliar screening method for soil-grown plants has been developed and tested. This new selection method was demonstrated to be a robust, high throughput approach for identifying transgenic *Arabidopsis*. Several novel vectors utilizing the *sul*I selectable marker have been constructed and shown to be useful for generating transgenic *Arabidopsis *plants. This new screening strategy and these useful vector tools, combined with the chemical stability and cost effectiveness of sulfonamide-based plant selection provide a desirable alternative to the existing transformation and selection methods. The plasmid vectors are available upon request and may be freely altered and/or redistributed for research purposes.

## Competing interests

The authors declare that they have no competing interests.

## Authors' contributions

JGT and RT conceived the project and directed the research. Vector design and construction was performed by JGT, MG and MC. Transgenic plant generation and characterization was performed by JS, JGT, MG and MC. Development and testing of the novel screening method was performed by JGT and JS. RT and JGT constructed the figures and wrote the manuscript with input from all of the authors. Each author reviewed and approved the final version of the manuscript.

## Supplementary Material

Additional file 1 **Demonstration of the use of *sul*I selection with plant lines containing other selectable markers**. *Arabidopsis *plants homozygous for both *npt*II and *hpt*II selection genes were crossed to lines containing pCS4-BASK (*sul*I). (A) F_1 _seeds (line 1.9) screened on MS media containing sulfadiazine (5 mg/L), hygromycin (10 mg/L) and kanamycin (50 mg/L) with Col-0 wild type as control. (B) PCR verification of T_2 _seeds from transgenic lines 1.9 and 6.13 containing the *sul*I, *hpt*II and *npt*II selection cassettes. The dash (-) indicates a water negative control, and the plus (+) indicates a plasmid positive control. A size standard labeled in kilobase pairs (kb) is shown. Click here for file
