# Supplementary material for: Novel sulI binary vectors enable an inexpensive foliar selection method in Arabidopsis
Source: BMC Res Notes. 2011 Mar 2;4:44. doi: 10.1186/1756-0500-4-44 (PMC3060128; doi:10.1186/1756-0500-4-44)
Supplement: Additional file 1 — Demonstration of the use of sulI selection with plant lines containing other selectable markers. Arabidopsis plants homozygous for both nptII and hptII selection genes were crossed to lines containing pCS4-BASK (sulI). (A) F1 seeds (line 1.9) screened on MS media containing sulfadiazine (5 mg/L), hygromycin (10 mg/L) and kanamycin (50 mg/L) with Col-0 wild type as control. (B) PCR verification of T2 seeds from transgenic lines 1.9 and 6.13 containing the sulI, hptII and nptII selection cassettes. The dash (-) indicates a water negative control, and the plus (+) indicates a plasmid positive control. A size standard labeled in kilobase pairs (kb) is shown. [file 1756-0500-4-44-S1.PDF]

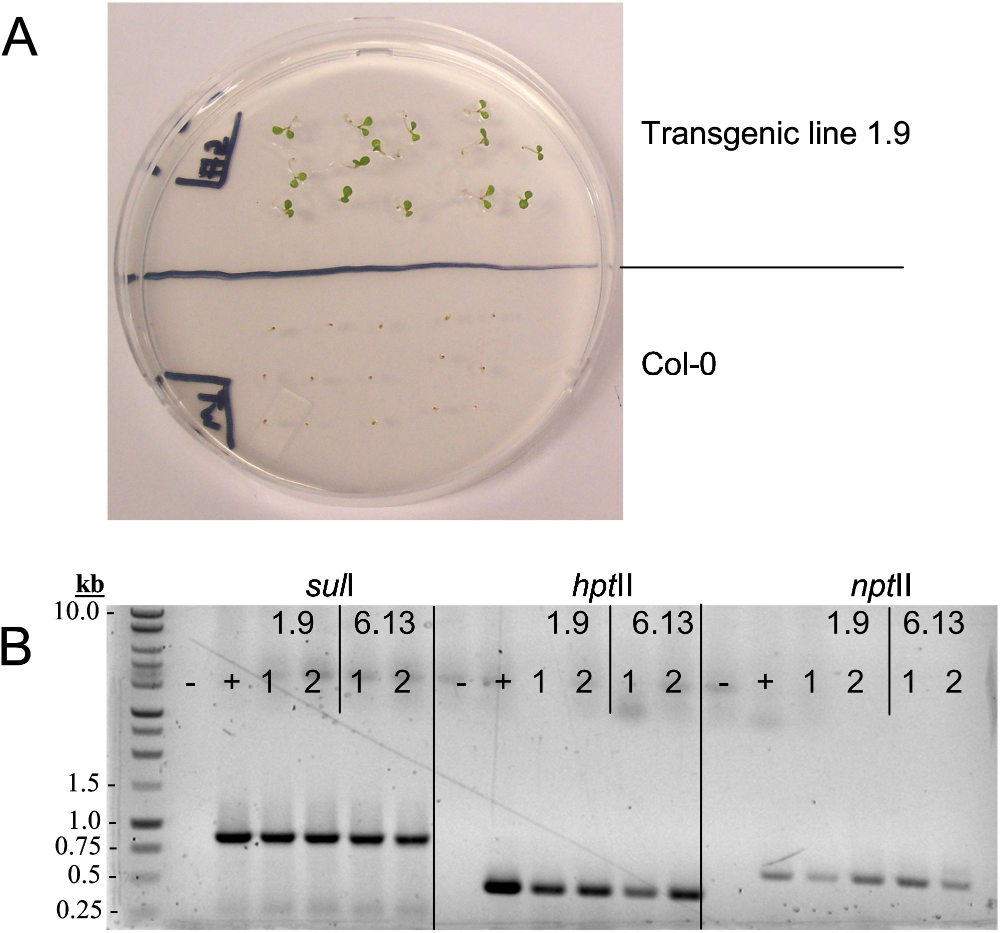

Additional file 1. Demonstration of the use of *sul*I selection with plant lines containing other selectable markers. *Arabidopsis* plants homozygous for both *npt*II and *hpt*II selection genes were crossed to lines containing pCS4-BASK (*sul*I). (A) F_1 seeds (line 1.9) screened on MS media containing sulfadiazine (5 mg/L), hygromycin (10 mg/L) and kanamycin (50 mg/L) with Col-0 wild type as control. (B) PCR verification of T_2 seeds from transgenic lines 1.9 and 6.13 containing the *sul*I, *hpt*II and *npt*II selection cassettes. The dash (-) indicates a water negative control, and the plus (+) indicates a plasmid positive control. A size standard labeled in kilobase pairs (kb) is shown.
